# Physical health behaviours and health locus of control in people with schizophrenia-spectrum disorder and bipolar disorder: a cross-sectional comparative study with people with non-psychotic mental illness

**DOI:** 10.1186/1471-244X-11-104

**Published:** 2011-06-24

**Authors:** Kurt Buhagiar, Liam Parsonage, David PJ Osborn

**Affiliations:** 1 Department of Mental Health Sciences, University College London Medical School, Rowland Hill Street, London NW3 2PF, UK

**Keywords:** attitudes, cardiovascular disease, health locus of control, physical health, severe mental illness

## Abstract

**Background:**

People with mental illness experience high levels of morbidity and mortality from physical disease compared to the general population. Our primary aim was to compare how people with severe mental illness (SMI; i.e. schizophrenia-spectrum disorders and bipolar disorder) and non-psychotic mental illness perceive their: (i) global physical health, (ii) barriers to improving physical health, (iii) physical health with respect to important aspects of life and (iv) motivation to change modifiable high-risk behaviours associated with coronary heart disease. A secondary aim was to determine health locus of control in these two groups of participants.

**Methods:**

People with SMI and non-psychotic mental illness were recruited from an out-patient adult mental health service in London. Cross-sectional comparison between the two groups was conducted by means of a self-completed questionnaire.

**Results:**

A total of 146 people participated in the study, 52 with SMI and 94 with non-psychotic mental illness. There was no statistical difference between the two groups with respect to the perception of global physical health. However, physical health was considered to be a less important priority in life by people with SMI (OR 0.5, 95% CI 0.2-0.9, *p *= 0.029). There was no difference between the two groups in their desire to change high risk behaviours. People with SMI are more likely to have a health locus of control determined by powerful others (*p *< 0.001) and chance (*p *= 0.006).

**Conclusions:**

People with SMI appear to give less priority to their physical health needs. Health promotion for people with SMI should aim to raise awareness of modifiable high-risk lifestyle factors. Findings related to locus of control may provide a theoretical focus for clinical intervention in order to promote a much needed behavioural change in this marginalised group of people.

## Background

People with mental illness experience excess morbidity and mortality from physical disease when compared with the general population [1-3]. Those suffering from severe mental illness (SMI), namely schizophrenia-spectrum disorders and bipolar disorder, have notably higher morbidity and mortality rates resulting from coronary heart disease (CHD) and stroke [4-7]. Their mortality rate directly linked to CHD is even greater than that arising from suicide [8]. Evidence further suggests that people with SMI may have a higher risk of mortality from natural causes compared with those suffering from non-psychotic mental illness including unipolar depression [9-13]. It is therefore not surprising that The National Institute for Clinical Excellence makes special emphasis on the importance of monitoring the physical health of people with SMI and research into appropriate interventions [14].

A number of factors may explain this increased burden of physical ill-health in people with SMI, including smoking, dietary habits, socioeconomic deprivation, co-morbid substance misuse disorders and anti-psychotic medication [8,15]. People with SMI also have restricted access to good quality medical care, such that their physical problems often go undetected or undertreated [16] in contrast to people with non-psychotic mental illness, who are more likely to take the initiative to seek medical care and make use of other health care services [9,17]. However, these factors may not wholly explain this increased adversity in people with SMI, suggesting a more intrinsic relationship between SMI and the development of physical illness [18]. In other words, people with SMI may have unique physical health risk factors over and above those associated with psychological and socioeconomic adversities common to people with mental illness at large.

We also know that people with SMI have poorer knowledge about physical activity, dietary habits and chronic physical problems compared with both people from the general population [19] and those with non-psychotic mental illness [18]. It has additionally been suggested that some people with SMI, notably those with schizophrenia, may have higher thresholds for pain sensitivity [20], further intensified by the analgesic effect of anti-psychotic medications [21]. This may subsequently preclude them from seeking medical care during the earlier stages of illness. Finally, people with SMI often have diminished insight into their mental health - a quality that is characteristically different from people with non-psychotic mental illness [22], and which may extend into the level of insight encompassing their physical health [12]. For instance, poor diet and exercise were described in people with SMI long after the psychotic symptoms had subsided [23]. Given the combination of these factors, it is possible that they may also prioritise their physical health differently and exhibit different levels of motivation to change high-risk behaviours related to CHD and associated disorders, such as smoking, lack of exercise and poor diet compared with people with non-psychotic mental illness.

Prochaska and DiClemente [24] propose that the ability to initiate behavioural change is dependent on several successive factors: an initial awareness of the harm caused to health by a specific behaviour, a subsequent desire to change this behaviour, and finally the successful actualisation of this change in behaviour. An intricately related construct to this model of behavioural change is Rotter's locus of control: a person's belief about the extent to which they can exert control over events that affect them [25]. Hence, according to this social learning theory, a person will embark on goal-oriented behaviour only if they are aware of the specific reinforcers available to them and if they believe that their behavioural change will lead to these reinforces in a particular situation [26]. With respect to their health, a person will seek to embark on health-related behavioural change if they both value their health and believe that any behavioural change will improve their health. People with a high internal locus of control feel more empowered to bring about this behavioural change independently, whereas those whose locus of control is located in powerful others or in chance (external locus of control) feel less empowered to bring about such behavioral change [26].

Given the evidence suggesting different health outcomes for people with SMI compared to those with non-psychotic mental illness, it would be important to elucidate any variations between groups of people with different mental illness in how they perceive their general physical health and how health locus of control may contribute to these perceptions. We are not aware of previous studies that have explored these factors in people with SMI compared to people with non-psychotic mental illness. Nevertheless, acquiring some understanding about these qualities is likely to be pivotal in planning a focus of clinical intervention with respect to health education packages and prophylactic measures that may improve the long-term outcomes, particularly those of people with SMI who may be at higher risk of physical health burden.

## Aims and objectives

We aimed to compare the physical health behaviours in a sample of people with SMI, our group of primary interest, compared to a sample of people with non-psychotic mental illness within a secondary care out-patient setting. The primary objectives of the study were to explore any differences between people with SMI and those with non-psychotic mental illness with respect to their:

(i) Perception of their overall physical health;

(ii) Prioritisation of their physical health in relation to other basic everyday needs;

(iii) Perception of barriers to improving their physical health;

(iv) Motivation to change modifiable risk factors for CHD, namely smoking, poor diet and poor exercise.

Our secondary aim was to investigate the potential contribution of health locus of control to these findings.

## Methods

This was a cross-sectional comparative study in a secondary care mental health service based in North London which we undertook in order to address various preliminary questions regarding a number of behaviours and attitudes towards physical health in people with SMI and non-psychotic mental illness. Ethical approval was obtained from the Camden and Islington Community Research and Ethics committee (Ref 05/Q0511/64). The study was also registered with the North Central London Research Consortium in accordance with guidance from the UK Department of Health Research Governance Framework for Health and Social Care.

We invited people with SMI and non-psychotic mental illness attending out-patient, care plan, and psychology clinics between January and June 2007 to participate in the study. A poster displayed in the waiting area of the clinic gave details of the study and the potential participants were asked if they would agree to be approached by a researcher (LP), who was present in the waiting area at specific set times. Those who agreed then received an information sheet about the study, and were able to ask questions to the researcher prior to taking part. The information sheet also included information material such as leaflets on how they could access services that could improve their physical health. Participants who provided written informed consent were then able to complete the questionnaire either on the day or take it away and return it at a later time. Instruction sheets on how to complete the questionnaire were included. Those who decided to complete the questionnaire on the day were provided with clipboards and pens, and returned the completed questionnaire in person to the researcher in a sealed envelope. Others who opted to take the questionnaire away were provided with a freepost envelope. It was therefore not possible to collect data on non-responders. Returned questionnaires were ultimately screened before data coding and entry so as to ensure that respondents who had been recruited did in fact meet the inclusion criteria.

We included participants if they were between the ages of 18-65 years and had a diagnosis of SMI (schizophrenia, schizoaffective disorder, bipolar disorder or other non-organic psychotic illness) or non-psychotic mental illness (unipolar depression, anxiety disorders or personality disorders) as established by their treating clinicians. Participants were subsequently divided into two groups: an "exposed group" with SMI and a comparison group without SMI. We deliberately opted to include people with non-psychotic mental illness as our comparison group as opposed to individuals from the general population on the basis that this would provide us with a unique opportunity to determine whether our outcomes of interest have specific correlations with SMI, rather than merely with mental illness at large. Participants were excluded if they were too unwell to take part in the study, or had a diagnosis of dementia, other organic brain disorders or an eating disorder (the latter due to possible distorted perceptions regarding diet and weight loss).

We collected data on age, gender, self-reported smoking status and a number of socioeconomic and demographic variables. Participants self-reported their psychiatric diagnosis, which was then cross-checked independently by two of the authors (LP and DPJO) with their pre-established ICD-10 [27] diagnosis documented in their medical case-notes. As all the components of the questionnaire in the study were self-reported, we did not ascertain the formal diagnosis by means of assessment schedules.

Participants completed the following questionnaires:

### (i) General physical health

We asked participants to rate their overall physical health in two ways. Firstly, they were asked to score their general health on a five-point Likert scale, a widely recognised method utilised in research involving self-rating of health [28]. Secondly, we asked participants to estimate their ten-year risk of suffering myocardial infarction, similarly on a five-point Likert scale.

### (ii) Health and lifestyle questionnaire

Motivation to change risk behaviours (smoking, poor diet and lack of exercise) was assessed with a "health and lifestyle questionnaire" that had been developed in a major study to assess attitudes towards cardiovascular risk factors in the general population [29]. For the purposes of our study, we adapted this questionnaire to include an additional final question related to the actualisation of behavioural change. Participants were asked: whether they were concerned about the physical health risks arising from these lifestyle behaviours; whether they desired to change their current behaviours; whether they had made a serious attempt to modify this behaviour in the previous year; and whether they were successful in bringing about behavioural change (adaptation).

### (iii) Attitudes towards physical health

To assess the relative importance of physical health for participants, we derived a number of basic everyday needs (including physical and mental health) from the Camberwell Assessment of Need questionnaire [30]. We then asked participants to select four items they deemed to be the most important to their lifestyle. We also asked participants to select four items they perceived to be the greatest barriers to improving their physical health.

### (iv) Multidimensional Health Locus of Control

To measure health locus of control we employed the Multidimensional Health Locus of Control (MHLC) scale [31]. This is a well validated scale that determines the degree to which a person perceives internal locus of control, powerful others and chance (the latter two, collectively referred to as "external locus of control") as being influential to their personal health status. The scale consists of 18 items and produces a score for the three subscales.

## Data analysis

We conducted data analysis using SPSS for Windows version 17.0 (SPSS Inc., Chicago, IL). We employed bivariate analysis to identify any significant differences between the two groups with respect to socio-economic variables. We used chi-square tests to establish any differences between the two groups with respect to lifestyle behaviours and motivation to modify these behaviours and calculated unadjusted odds ratios and confidence intervals. We initially explored association between our participants and the other main outcomes of interest (priorities in life, barriers to improving physical health and health locus of control) by means of bivariate analysis. The results of this analysis then provided us with a guide for inclusion of co-variates in subsequent multivariate analysis. On the basis of their statistically significant association with SMI on bivariate analysis, we used employment, education, and duration of illness in the model. We also included age and gender *a priori *in this analysis in view of their potential confounding effect on the association between mental illness and health behaviours. We used binary logistic regression for dichotomous outcomes and linear multiple regression for continuously distributed variables, i.e. health locus of control sub-scales.

## Results

### Response rates

Of 245 people attending the clinics who were approached to take part in the study, 146 (59.6%) completed the questionnaires. In total, 52 participants (35.6%) had a diagnosis of SMI whereas 94 (64.4%) suffered from non-psychotic mental illness. Complete and valid data were available for all respondents.

### Characteristics of participants

The demographic and socioeconomic characteristics of the two groups are described in Table 1. Of 52 people with SMI, 34 (65.4%) had schizophrenia, 4 (7.7%) had schizoaffective disorder and 14 (26.9%) had bipolar affective disorder. In the group with non-psychotic mental illness, 65 (69.1%) had unipolar depression, 14 (14.9%) had an anxiety disorder, and 15 (16.0%) had a primary diagnosis of personality disorder. Amongst participants with SMI, 46 (88.5%) reported the correct clinical diagnosis established by their clinical team, while 88 (93.6%) participants in our comparison group reported the correct pre-established diagnosis (*p *= 0.348).

**Table 1:** Demographic and socio-economic variables associated with severe mental illness (SMI).

Variable	SMI (*n *= 52) *n *(%)	Non-psychotic mental illness (*n *= 94) *n *(%)	χ^2^	*P*
Gender				
Male	28 (53.8)	32 (34)	5.24	0.02
Female	24 (46.2)	62 (66)		
Age, mean (SD)	43.8 (±10.7)	42 (±13.6)	-	0.424^a^
Employment				
Unemployed^b^	31 (59.6)	12 (12.8)	35.37	<0.001
Employed^c^	21 (40.4)	82 (87.2)		
Ethnicity (self-defined)				
White	41 (78.8)	84 (89.4)	3	0.083
Black or minority	11 (21.2)	10 (10.6)		
Education				
School only	30 (58.8)	34 (36.2)	6.88	0.009
Further education	21 (42.2)	60 (63.8)		
Duration of illness since diagnosis, years				
<1	0 (0)	5 (5.3)		
1-5	14 (26.9)	51 (54.3)	16.35	<0.001
6-10	38 (73.1)	37 (39.4)		
>10	0 (0)	1 (1.1)		

^a ^*t*-test

^b ^Includes those in receipt of state of benefits

^c ^Includes retired, student and homemaker status

### Physical health outcomes

The perception of overall physical health was broadly similar between the two groups, with 27 participants with SMI (51.9%) and 50 participants with non-psychotic mental illness (53.2%) describing it as being "excellent", "very good" or "good" (OR 0.8, 95% CI 0.4-1-6, *p *= 0.887). The two groups of participants also reported similar responses with respect to their perceived likelihood of suffering from myocardial infarction in the next ten years: 36 participants with SMI (69.2%) and 63 participants with non-psychotic mental illness (67.0%) considered the event as being "unlikely" or "very unlikely" to happen to them (OR 1.1, 95% CI 0.5-2.3, *p *= 0.920).

### Lifestyle factors and behavioural change

Table 2 summarises the perceptions of physical health risk associated with the three lifestyle factors of interest, namely smoking, exercise and diet, as well as the desire to change, attempts to change and success in changing these behaviours. People with SMI were significantly more likely to smoke (OR 4.0, 95% CI 2.0-8.3, p < 0.001). However, there was no statistical difference between the two groups with respect to their level of perceived physical health risk arising from smoking and subsequent motivation to change, attempts to change and success in changing this behaviour. Nearly all of our participants reported not getting enough exercise (SMI, n = 51, 98.1% vs. non-psychotic mental illness, n = 89, 94.7%; OR 2.9, 95% CI 0.3-25.2, p = 0.326) and having a poor diet (SMI, n = 51, 98.1% vs. non-psychotic mental illness, n = 89, 94.7%; OR 2.9, 95% CI 0.3-25.2, p = 0.326). There was no statistical difference between the two groups with respect to subjective perception about their diet and lack of exercise and their effect on physical health risks. Similarly, there was no difference in the groups' desire to change and success in changing these two lifestyle factors. However, people with SMI were much less likely to have attempted to increase their levels of exercise during the past year (OR 0.2, 95% CI 0.01-0.6, p = 0.005).

**Table 2:** Motivation to change lifestyle behaviours in people with severe mental illness (SMI) (*n *= 52) and people with non-psychotic mental illness (*n *= 94).

Lifestyle Behaviour	Report behaviour		OR (95% CI)	Concerned about behaviour		OR (95% CI)	Want to change behaviour		OR (95% CI)	Tried to change behaviour		OR (95% CI)	Successfully changed behaviour		OR (95% CI)
	
	SMI *n* (%)	Non-SMI* n (%)		SMI *n* (%)	Non-SMI* n (%)		SMI *n* (%)	Non-SMI* n (%)		SMI *n* (%)	Non-SMI* n (%)		SMI *n* (%)	Non-SMI* n (%)	
Smoking	34 (65.4)	30 (31.9)	4.0 (2.0-8.3) ***P *< 0.001**	28 (82.4)	26 (86.7)	0.7 (0.2-2.8) *p *= 0.897	24 (85.7)	18 (69.2)	2.7 (0.7-10.3) *p *= 0.207	15 (62.5)	12 (66.7)	0.8 (0.2-3.0) *p *= 0.963	1 (6.7)	1 (8.3)	0.8 (0.04-0.03) *p *= 0.565
Lack of exercise	51 (98.1)	89 (94.7)	2.9 (0.3-25.2) *p *= 0.326	34 (66.6)	60 (67.4)	1.4 (0.7-2.9) *p *= 0.396	33 (97.0)	51 (85.0)	5.8 (0.7-48.1) *p *= 0.141	13 (39.4)	37 (72.5)	0.2 (0.01-0.6) ***p *= 0.005**	5 (41.7)	15 (40.5)	0.7 (0.2-2.6) *p *= 0.862
Poor diet	51 (98.1)	89 (94.7)	2.9 (0.3-25.2) *p *= 0.326	20 (39.2)	29 (32.6)	1.3 (0.6-2.7) *p *= 0.533	15 (75.0)	26 (89.7)	0.3 (0.07-1.6) *p *= 0.281	7 (46.7)	16 (61.5)	0.5 (0.2-2.0) *p *= 0.548	4 (57.1)	10 (62.5)	0.8 (0.1-4.9) *p *= 0.824

*"Non-psychotic mental illness" has been abbreviated as "Non-SMI" in order to accommodate spatial restrictions;

OR: Odds ratio for SMI

### Priorities in life and barriers to improving physical health

Data are summarised in Table 3. Participants with SMI were less likely to rank physical health (OR 0.5, 95% CI 0.2-0.9, *p *= 0.029), accommodation (OR 0.4, 95% CI 0.2-0.9, *p *= 0.022) and friends and family (OR 0.2, 95% CI 0.1-0.6, *p *= 0.006) as one of their top four priorities. However, the difference between the two groups with respect to accommodation did not remain significant following adjustment for confounding variables (adjusted OR 0.5, 95% CI 0.2-1.0, *p *= 0.056). On the other hand, people with SMI were more likely to regard their mental health as a main priority, after adjustment for confounding variables (adjusted OR 2.2, 95% CI 1.0-4.7, *p *= 0.049). Regarding barriers to improving physical health, there were no statistical differences between the two groups on any of the twelve variables presented. However, both groups of participants equally considered their mental health to be the greatest barrier to improving their physical health.

**Table 3:** Priorities in life and barriers to giving priority to physical health ranked in the top four by people with severe mental illness (SMI) and with non-psychotic mental illness.

Variable	SMI n = 52 *n *(%)	Non-psychotic mental illness n = 94 *n *(%)	Unadjusted OR (95% CI)	*χ2 *(*P)*	**Adjusted OR**^**a**^ (95% CI)	Adjusted *P*
***Priorities***						
Accommodation	29 (55.8)	70 (74.5)	0.4 (0.2-0.9)	5.3 (**0.022)**	0.5 (0.2-1.0)	0.056
Daytime activities	16 (30.8)	13 (13.8)	2.8 (1.2-6.4)	5.8 **(0.016)**	0.5 (0.2-1.2)	0.109
Education	10(19.2)	17 (18.1)	1.1 (0.5-2.6)	0.0 (0.864)	1.5 (0.6-4.2)	0.388
Friends and family	37 (71.2)	86 (91.5)	0.2 (0.1-0.6)	10.0 **(0.002)**	0.2 (0.1-0.7)	**0.006**
Looking after home	6 (11.5)	11 (11.7)	1.0 (0.3-2.8)	0.0 (0.976)	1.1 (0.4-3.6)	0.830
Mental health	35 (67.3)	61 (64.9)	1.1 (0.5-2.3)	0.1 (0.769)	2.2 (1.0-4.7)	**0.049**
Money	19 (36.5)	27 (28.7)	0.7 (0.3-1.4)	0.9 (0.331)	1.6 (0.7-3.6)	0.232
Physical health	27 (51.9)	66 (70.2)	0.5 (0.2-0.9)	4.8 **(0.029)**	0.4 (0.2-0.9)	**0.018**
Transport	3 (5.8)	1 (1.1)	5.7 (0.6-56.2)	2.7 (0.136)	4.8 (0.4-53.1)	0.198
Work	13 (25.0)	34 (36.2)	1.7 (0.8-3.6)	2.0 (0.169)	0.6 (0.3-1.3)	0.176
***Barriers***						
Accommodation	7 (13.5)	7 (7.4)	1.9 (0.6-5.9)	1.3 (0.243)	1.3 (0.4-4.5)	0.638
Difficulty going out	16 (30.8)	24 (25.5)	1.3 (0.6-2.7)	0.5 (0.497)	1.3 (0.6-2.9)	0.528
Do not know who to ask	4 (7.7)	9 (9.6)	0.8 (0.2-2.7)	0.1 (0.703)	0.7 (0.2-2.4)	0.536
Embarrassed	3 (5.8)	11 (11.7)	0.5 (0.1-1.7)	1.5 (0.253)	0.7 (0.2-2.8)	0.591
Family and friends	10 (19.2)	23 (24.4)	0.7 (0.3-1.7)	0.5 (0.470)	0.8 (0.3-2.0)	0.661
Mental health	26 (50.0)	44 (46.8)	1.1 (0.6-2.2)	0.1 (0.712)	1.0 (0.5-2.1)	0.959
Money	7 (13.5)	22 (23.4)	0.5 (0.2-1.3)	2.2 (0.154)	0.5 (0.2-1.4)	0.201
No appointments	5 (9.6)	6 (6.4)	1.6 (0.5-5.4)	0.5 (0.481)	2.0 (0.5-7.7)	0.332
No one listens	7 (13.5)	4 (4.3)	3.5 (1.0-12.6)	3.9 (0.055)	2.6 (0.6-10.8)	0.183
No one to ask	4 (7.7)	9 (9.6)	0.8 (0.2-2.7)	0.1 (0.703)	0.7 (0.2-2.8)	0.666
Not concerned	5 (9.6)	21 (22.3)	0.4 (0.1-1.0)	4.0 (0.061)	0.4 (0.1-1.1)	0.074
Not worried	12 (23.1)	21 (22.3)	1.0 (0.5-2.3)	0.0 (0.919)	0.9 (0.4-2.2)	0.838

^a ^Adjusted for age, gender, duration of illness and employment

### Locus of control

Participants with SMI had statistically significant higher scores on the MHLC for powerful others (mean score, SMI: 24.25 vs. non-psychotic mental illness: 17.71, *p *< 0.001) and chance (mean score, SMI: 20.62 vs. non-psychotic mental illness: 17.74, *p *= 0.006), but no difference in the scores for an internal locus of control when compared to people with non-psychotic mental illness (mean score, SMI: 23.52 vs. non-psychotic mental illness: 24.17, *p *= 0.536). There was negligible change in results following adjustment for potential confounders (powerful others, *p *< 0.001; chance, *p *= 0.037; internal locus of control, *p *= 0.768).

## Discussion

Participants with SMI rated their global physical health and their perceived risk of suffering from a myocardial infarction similarly to people with non-psychotic mental illness. Indeed, less than half of them expressed concern about the possibility of having sub-optimal physical health or that they may be at risk of developing serious physical health illnesses. A growing body of research postulates that SMI itself may be a risk factor for CHD, stroke and diabetes [6,12,15,32] in excess of the risks carried by the general population, and to a lesser extent in excess of those with people with non-psychotic mental illness [9-13]. Nevertheless people with SMI may not be entirely aware of these increased physical health risks. This finding is consistent with our previous work suggesting that people with SMI are likely to have poor level of knowledge regarding specific risks factors for CHD [18]. Similar findings have been reported with respect to the knowledge about diabetes amongst people with SMI and co-occurring type 2 diabetes compared to people with non-psychotic mental illness as well as the general population [19].

A more surprising finding is the relatively optimistic judgement about their physical health demonstrated by participants with non-psychotic mental illness in our sample, despite that people with anxiety and depression have consistently been shown to have higher levels of physical health disability [33,34]. The level of neuroticism inherent to these illnesses is also associated with excess reporting of somatic symptoms [35] and a propensity to seek medical assistance for physical symptoms [9,17]. At the same time, it also known that people in the recovery phase from depression and anxiety demonstrate less physical disability [33], which may also extend to curtailed knowledge and apprehension about physical health. It is therefore possible that our sample may have contained a large proportion of participants in the recovery phase of their illness, in addition to those with personality disorder, diluting the concern about physical health that would have otherwise been expected from this participant group.

People with SMI in our study do not consider their physical health to be one of the main priorities in their life. On the other hand, given the chronic nature and severity of their mental illness, they may understandably reserve a greater proportion of their energy to attempt to optimise their mental health. In other words, people with SMI may recognise the great burden that their mental illness can impose on their quality of life [36], while overlooking the potential contribution of their physical health to this impaired quality of life. In contrast, given the preoccupation with physical illness usually demonstrated by people with non-psychotic mental illness [34], our participants in this sub-group viewed their physical health as one of their greatest priorities. This finding is sharply incongruent with our other result suggesting lower than expected levels of awareness about physical health by this group of individuals. A plausible explanation could be that the broader and more in-depth nature of the questionnaire utilised to capture this aspect of behaviour was more successful at eliciting physical health concern in these people.

People with SMI and non-psychotic mental illness equally view their mental illness as a major barrier to improving their physical health. Our sample of participants in the latter category was drawn from a secondary care out-patients service, where the degree of psychiatric morbidity is likely to have been at the more severe end of the illness spectrum. This may have been a major contribution our finding. We were unable to explicitly bring to light any other specific barriers to improving physical health in either group of participants. A recent thorough narrative review of incentives and barriers to healthy living or lifestyle interventions for people with SMI did highlight the relatively sparse research specifically designed to address these issues [37]. However, identified barriers include psychiatric symptoms, in line with our results, as well as adverse effects of medications and negative attitudes of healthcare professionals.

Similar to findings from previous studies [38], people with SMI were also more likely to be smokers, contributing to their risks of physical disease. Additionally, all but one participant reported lack of exercise and poor diet. In fact, in a previous UK study amongst people with SMI, only one-third of participants with SMI reported eating at least one fruit a day [39]. Physical inactivity and poor diet in the form of low fibre and high saturated fat intake have already been postulated to partly explain the increased CHD-risk associated with SMI irrespective of medication treatment and socio-economic variables [6]. This combination of low priority given to their physical health, lack of awareness about increased risk to physical health and increased health-related risk behaviours, poses a significant challenge to improving the physical health in this population group. Signs of early CHD and other related problems such as hypertension and blood lipid abnormalities can often go unnoticed unless directly monitored [40]. As those who suffer with SMI are unaware of their increased physical health risks, efforts need to be made in order to increase the knowledge amongst people with SMI related to these risks and subsequently improve uptake of health monitoring tests. Additionally, findings from other studies suggest that people with SMI and chronic somatic disease are likely to have an even poorer quality of life than people with SMI alone [41]. All of these factors therefore highlight the importance of implementing early behavioural lifestyle interventions aimed at improving physical health outcomes for this group of people. Evidence from studies amongst people with schizophrenia also suggests that these interventions can indeed be effective, for instance in reducing antipsychotic-induced weight gain [42].

We did not evaluate cognitive functioning in our participants. However, previous work has shown that the knowledge about diabetes in people with SMI may be directly correlated with their level of cognitive ability [19]. Strategies aimed at increasing the awareness of the physical health risks in people with SMI should therefore also pay recognition to these cognitive deficits, and ensure that cognitive loads are maintained to a minimum.

Lack of motivation as a negative symptom of psychotic illnesses could be implicated in the poor physical health of people with SMI, and earlier small studies evaluating motivation to exercise seem to imply so [37,43,44]. However our findings suggest that there is no difference in people with SMI from those with non-psychotic mental illness with respect to their desire to change high-risk lifestyle behaviours, namely smoking, poor diet and lack of exercise. Poor awareness may therefore be a key barrier to improving physical health in people with SMI rather than a lack of motivation *per se*. In fact, our previous work has shown that people with SMI are willing to participate in cardiovascular screening programmes based in primary care, if invited to do so, with participation rates being similar to those from community-based populations [45]. Moreover, a recent study evaluating an intervention targeted at increasing exercise in people with SMI revealed that people with SMI are keen to participate in these programmes provided that they are acceptable and carefully designed to meet the specific needs of this population group [46].

It has long been well-established that people with depression and anxiety disorders [47], as well as those with personality disorders [48] demonstrate greater externality in their locus of control compared to non-psychiatric populations. However, our participants with SMI exhibited even greater external health locus of control than people with non-psychotic mental illness, as evidenced by the results of the "powerful others" and "chance" subscales of the MHLC. People with more chronic forms of psychosis have already been to shown to be more likely to report having less control over their mental illness and a more external locus of control than people with less chronic forms of SMI [49]. A smaller study also showed that people with schizophrenia (*n *= 22) have higher scores on external health locus of control measures compared with population norms [50]. We are not aware of previous studies that have explored locus of control in people with SMI in relation to people with non-psychotic mental illness. This high external locus of control is likely to be a reflection of the patients' feelings that their illness may be outside their control given its occasional unpredictability, which may additionally extend to their perceived level of control over their physical health. Ultimately, it may also indicate that health professionals are in a good position of exerting a high level influence on people with SMI with regards to their physical health and this fact could be used advantageously when designing interventions directed to improve physical health. Greater awareness of this finding will also remind clinicians to work towards empowering their patients.

### Limitations

We were unable to determine the profile of those who declined to take part in the study. It is possible that those who did not participate preferred not to take part as a result of strong beliefs about their physical health or perhaps poor physical health and this could therefore have influenced our findings. However there is no reason to expect that this bias would apply differently to the two groups. We employed measures of overall health which are simple, have been used extensively and shown to have validity. However the questionnaires have not been specifically designed to be used amongst populations with mental illness. Overall there were enough participants in the study to give reliable results in the statistical analysis. However this study is likely to be underpowered with respect to results concerning motivation to change, which might limit the strength of these findings. Moreover, our study was based entirely on self-report measures, which limited the breadth and nature of data that could be collected, such as past psychiatric history, severity of illness and other clinical variables. Ideally, we should have also included a third group of participants from the general population as this would have made our findings even more robust. Nevertheless, our central objective was to explore whether people with SMI exhibit unique characteristics in their physical health behaviours and health locus of control compared with people with non-psychotic mental illness. Finally, it is also acknowledged that we addressed a wide range of questions, which may have precluded our study from having clear-cut and succinct objectives. However, this study was of a preliminary nature set against the prospect of addressing more tightly focused research questions in the near future, guided by the findings of the present study.

### Clinical Implications

This study raises important issues concerning the physical health needs of people with SMI. It continues to emphasise the importance of focusing on lifestyle issues for people with SMI in order for them to engender change that decreases the burden on their physical health. Rather than lack of motivation being a key factor in affecting physical health it appears that lack of awareness and a lack of prioritisation are the main obstacles to improving physical health in this population group. Furthermore, people with SMI are more likely to express greater externality in health locus of control compared with people with non-psychotic mental illness. Clinicians could therefore exploit this finding to help address lifestyle and physical health needs of these patients. Interventions should also aim to increase the awareness of healthcare professionals about the physical health needs of people with SMI. Evidence does suggest that behavioural lifestyle interventions are more likely to be taken up by people with SMI when the support of healthcare professionals is available in these interventions [37]. This will allow them to act more pro-actively in encouraging patients to participate in routine physical health assessments and prophylactic measures.

## Conclusions

Despite evidence for increased physical disease in people with SMI compared to people with non-psychotic mental illness and the general population, this group of individuals are likely to give little attention to their lifestyle and physical health needs. However, this may arise from impaired awareness of the implications of their risk behaviour rather than due to a lack of motivation. People with SMI appear to demonstrate even greater externality of health locus of control compared to people with non-psychotic mental illness. This finding could provide an important focus of clinical intervention, as it places healthcare professionals in a very favourable position to exert their influence by means of health promotion and active therapeutic interventions that reduce modifiable risk factors for physical disease and improve outcomes. Further research could investigate how specific clinical interventions could be implemented in order to provide a coherent healthcare service that straddles both physical and mental health needs of marginalised individuals with SMI.

## Competing interests

The authors declare that they have no competing interests.

## Authors' contributions

DPJO and LP conceived the idea and design of the study and helped draft the manuscript. LP collected the data. KB conducted the data analysis and interpretation and produced the initial manuscript draft. All authors read and approved the final manuscript.

## Pre-publication history

The pre-publication history for this paper can be accessed here:

http://www.biomedcentral.com/1471-244X/11/104/prepub
